# Stent Placement for Acute Superior Mesenteric Artery Occlusion Associated with Type B Aortic Dissection

**DOI:** 10.1155/2015/485141

**Published:** 2015-05-25

**Authors:** Kazushi Suzuki, Masashi Shimohira, Takuya Hashizume, Yuta Shibamoto

**Affiliations:** Department of Radiology, Nagoya City University Graduate School of Medical Sciences, Nagoya 467-0001, Japan

## Abstract

A 50-year-old man had a mesenteric ischemia related to superior mesenteric artery (SMA) occlusion associated with a type B aortic dissection. We decided to perform stent placement for the SMA and could avoid mesenteric ischemia. We think the stent placement in the SMA might be an option for the treatment of mesenteric ischemia caused by aortic dissection.

## 1. Introduction

Mesenteric ischemia is a life-threatening complication of acute type B aortic dissection. However, there are few reports on its endovascular repair. We present a case with bowel ischemia related to an acute type B dissection successfully undergoing stent placement for the superior mesenteric artery (SMA).

## 2. Case Presentation

**Figure 1:**
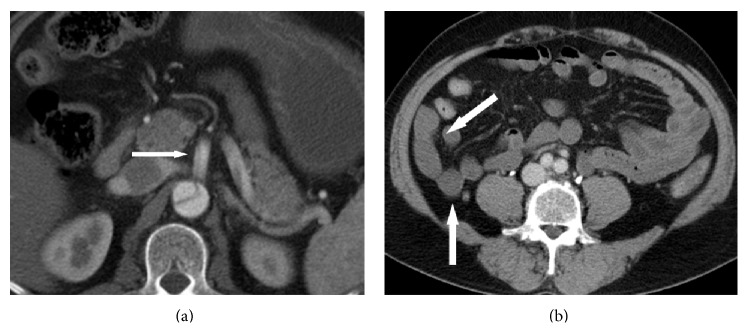
Contrast-enhanced CT shows (a) a type B aortic dissection, dissected SMA, and compressed true lumen (arrow) and (b) poor contrast enhancement of some of the small intestines (arrow).

**Figure 2:**
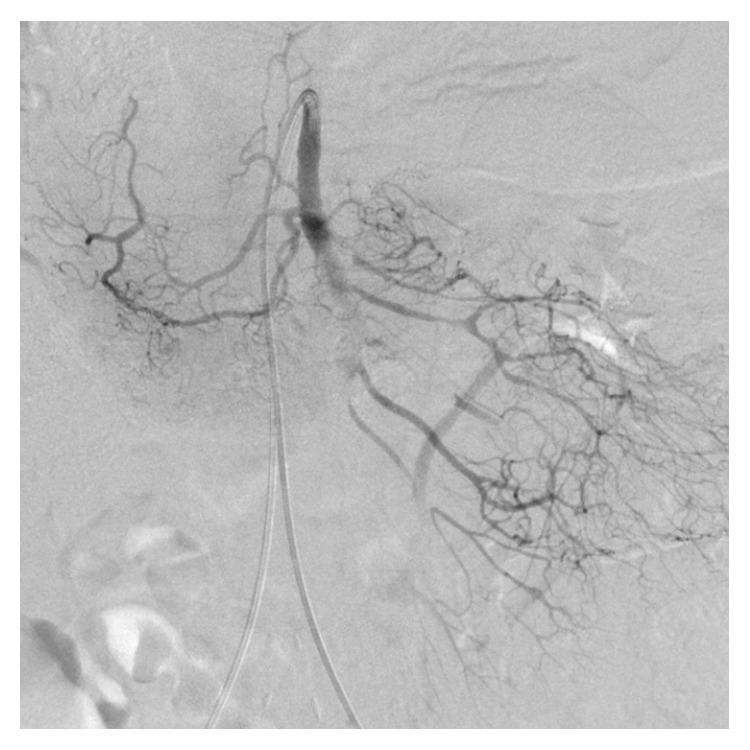
Angiography shows occlusion of the SMA with missing branches.

**Figure 3:**
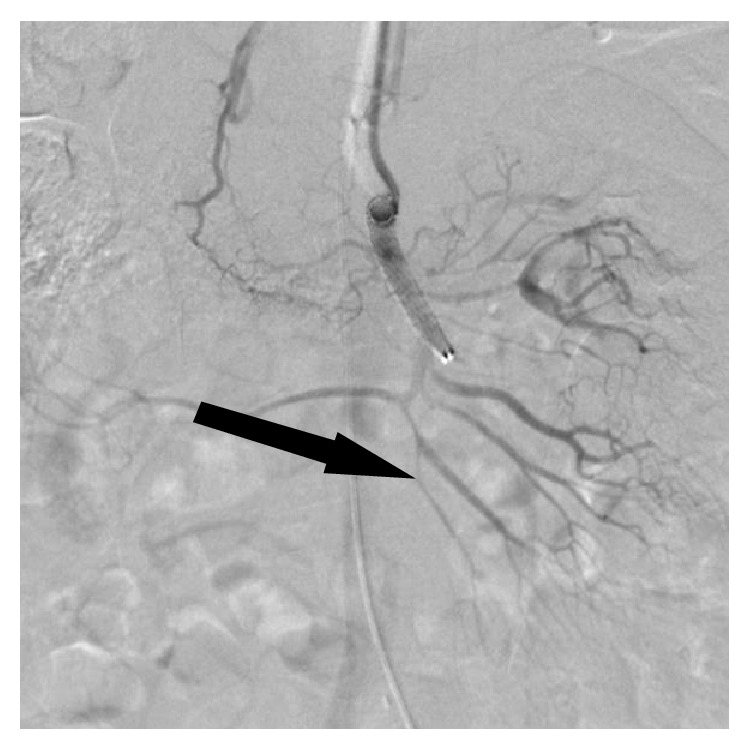
Angiography of the SMA after stent placement shows improved peripheral blood flow (arrow).

**Figure 4:**
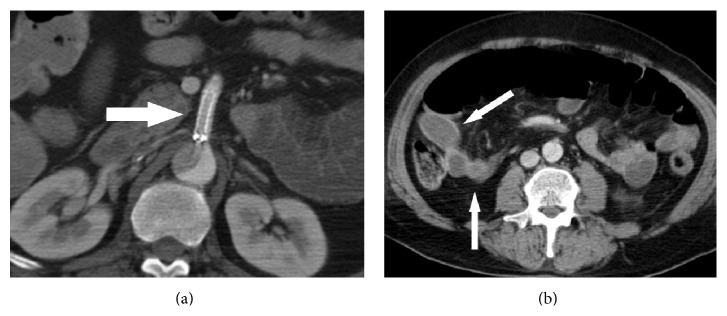
Contrast-enhanced CT shows (a) the patency of the stent placed in the SMA (arrow) and (b) the improvement of enhancement of the small intestine (arrow).

**Figure 5:**
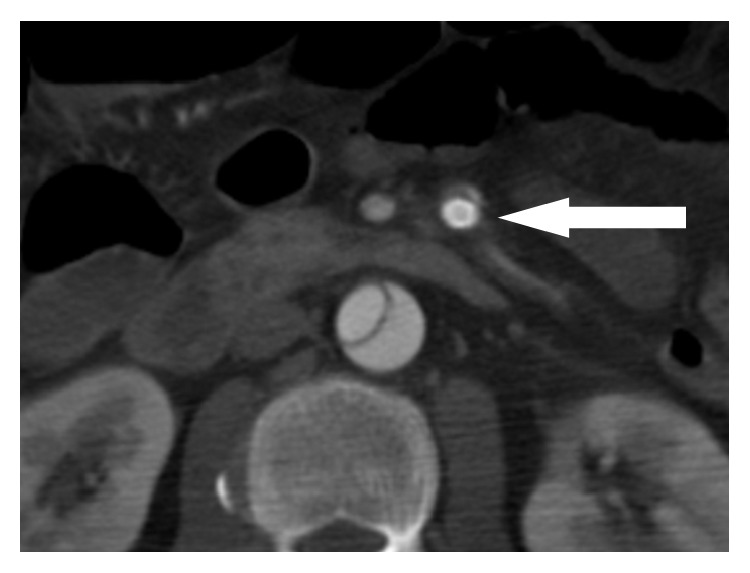
Contrast enhancement in the internal SMA stent was well maintained on contrast-enhanced CT at two weeks after the stenting.

A 50-year-old man suddenly felt a severe chest and back pain and came to our hospital by ambulance. He had a medical history of schizophrenia. However, cardiovascular risk factors had not been pointed out. There was no abnormality in laboratory data except for an increased white blood cell count (18000/*μ*L.). Pentazocine was used intravenously for his severe pain. Heparin or other antiplatelet drugs had not been administered. Contrast-enhanced computed tomography (CT) revealed a type B aortic dissection from the descending aorta to the left common iliac artery and narrowing of the true lumen of the abdominal aorta. According to this CT, the SMA originates partially from the true and partially from the false lumen. The true lumen was compressed (Figure 1(a)), and the intestinal tract was poorly enhanced (Figure 1(b)). Emergency angiography was performed from the right femoral artery using a 4 Fr sheath and a 4 Fr shepherd-hook catheter (Terumo, Tokyo, Japan) to evaluate the blood flow in the SMA. At this time, the catheter was confirmed to be in the true lumen by intravascular ultrasound (IVUS). The angiography showed occlusion of the SMA and some of small intestine had poor contrasting effect (Figure 2). So, the placement of a metallic stent in the true lumen of the SMA was considered. However, the angle of the SMA was so steep that it was difficult to insert the guidewire deeply from the right femoral artery. Therefore, it was then approached from the left brachial artery to the SMA using a 6 Fr multipurpose catheter (Terumo, Tokyo, Japan) and a 6 Fr sheath. The microguidewire could be inserted to the SMA deeply, and it was confirmed to be in the true lumen by IVUS again. A metallic stent (E-Luminexx Vascular Stent, Bard Peripheral Vascular, Inc., Tempe, AZ, USA) with a diameter of 6 mm and a length of 6 cm was then placed into the SMA. We decided to use this self-expandable stent because the abdomen is at a risk of strong compression by trauma. We considered that a self-expandable stent was better than a balloon-expandable stent because of its greater radial strength [1]. Thereafter, the peripheral blood flow of the SMA was improved (Figure 3). The condition of the intestine was checked by laparoscopy immediately; the color of the terminal ileum was slightly dark, but the serosal surface and peristalsis looked good, so it was observed. On the next day, contrast-enhanced CT revealed improvement of blood flow of the SMA (Figure 4(a)) and enhancement of the intestinal tract (Figure 4(b)). It was confirmed that the color of the terminal ileum became better by laparoscopy. Furthermore, this CT revealed the aortic dissection changed to a type A dissection, and total aortic arch replacement was performed immediately after the laparoscopy. Heparin (15000 units per day) was injected for 7 days, and thereafter two antiplatelet drugs (200 mg cilostazol and 200 mg aspirin) were prescribed as an anticoagulation therapy. Contrast effect of internal SMA stent had been well maintained in contrast-enhanced CT in two weeks after the stenting (Figure 5). There was no complication related with the stenting, but he had severe pneumonia and it took 3 months to discharge. Thereafter, there has been no finding of mesenteric ischemia during a 2-year follow-up period.

## 3. Discussion

Typically, type B dissection occurs in elderly hypertensive males and presents with abrupt onset of chest and/or back pain. Most patients with type B dissection do not present with hemodynamic instability, hypotension, or spinal cord and/or mesenteric ischemia, and even pulse deficit is uncommon [1]. The present case was not typical in the fact that the patient was relatively young and had no hypertension. Thus, it was difficult to speculate the cause of the type B dissection. However, the onset was typical, with abrupt back pain, and the dissection was suspected. At present, the preferred treatment for most patients with type B dissection is medical therapy, including the use of antihypertensive drugs and beta-blockers. Indications of surgical treatment include specific cases complicated by progression of dissection, impending rupture, refractory hypertension, localized false aneurysm, continued pain, and end-organ ischemia caused by compromise of the aortic branches [2].

However, Cambria et al. [3] reported that noncardiac vascular complications in the aortic dissection occurred in 33% of the patients, and in these patients the overall mortality rate (51%) was significantly higher than in those without such complications (29%). Although aortic rupture was the strongest correlate of mortality (90%), death specifically related to vascular occlusion was common when such occlusion occurred in the carotid, mesenteric, and renal circulation. The mesenteric ischemia was reported with less than 5% incidences [4]. However, the operative mortality rate for patients with this complication was reported to be as high as 88%, and death often resulted from the sequelae of mesenteric infarction [3]. Thus, the endovascular treatment seems important. Dake et al. [5] reported that, for treatment of the acute aortic dissection, the placement of endovascular stent graft across the primary entry tears was safe and effective. They also showed that the effect of this endovascular technique for the treatment of peripheral ischemic sequelae of aortic dissection was well established; restoration of the blood flow to compromised vessels and associated clinical improvements have been achieved in 92 to 100% of cases reported. In our country, however, only physicians who have a special license of aortic stent grafting can perform this procedure. Furthermore, the stent graft devices are not stored in hospital usually, and it takes some time to obtain them. It is generally considered that endovascular treatment should be done within 5 hours after the SMA trunk occlusion [6]. Thus, it can be performed only in limited situations. On the other hand, the stent placement in the SMA can be performed easily and quickly, and it is an established procedure for the SMA dissection [7, 8].

In our case, when it was diagnosed, 4.5 hours had passed after the onset. So, we performed the stent placement for the SMA, and as a result we could avoid resection of the small intestine. We think the stent placement in the SMA might be an option for the treatment of mesenteric ischemia caused by aortic dissection, especially in a situation including absence of a doctor with a sufficient skill of the aortic stent grafting, no aortic stent graft device in the hospital, and a long time needed for delivery of an aortic stent graft device to the hospital. However, the primary entry tear is not treated with this procedure, so it must be necessary to carefully observe the patient or to prepare for the aortic stent grafting.
